# Coreflooding data on nine sandstone cores to measure CO_2_ residual trapping

**DOI:** 10.1016/j.dib.2019.104249

**Published:** 2019-07-12

**Authors:** Hailun Ni, Maartje Boon, Charlotte Garing, Sally M. Benson

**Affiliations:** a Department of Energy Resources Engineering, Stanford University, Stanford, CA, USA; b Department of Geology, University of Georgia, Athens, GA, USA

**Keywords:** CO_2_ storage, Residual trapping, Multiphase flow, Coreflooding experiment

## Abstract

This data article provides detailed explanation and data on CO_2_/water coreflooding experiments performed on nine sandstone rock cores. Refer to the research article “Predicting CO_2_ Residual Trapping Ability Based on Experimental Petrophysical Properties for Different Sandstone Types” [1] for data interpretation. The reader can expect to find experimental conditions including temperature, pressure, fluid pair types, as well as flow rates. Furthermore, the raw CT images and the processed three-dimensional (3D) voxel-level porosity, permeability, and CO_2_ saturation maps for each of the nine sandstone samples are also supplied.

**Table:** Specifications Table

Subject area	*Climate science, geology, physics*
More specific subject area	*Multiphase flow in porous media, CO*_*2*_*residual trapping*
Type of data	*Table, figure, CT image, Matlab data file*
How data was acquired	*Medical CT scanner (General Electric Hi-Speed x-ray CT)*
Data format	*Raw and analyzed*
Experimental factors	*CO*_*2*_*/water drainage and imbibition experiments are conducted*
Experimental features	*CO*_*2*_*saturation, porosity, and permeability matrices are extracted*
Data source location	*Stanford, USA*
Data accessibility	Repository name: Mendeley Data Data identification number: https://doi.org/10.17632/wrgdmhyrps.2 Direct URL to data: https://data.mendeley.com/datasets/wrgdmhyrps/2
Related research article	H. Ni, M. Boon, C. Garing, and S. M. Benson, Predicting CO_2_ residual trapping ability based on experimental petrophysical properties for different sandstone types, Int. J. Greenh. Gas Control, 86 (2019) 158–176 [1].

**Table:** 

Value of the data • The CO_2_ saturation maps allow researchers to calculate and compare CO_2_ residual trapping relationships for different sandstone types, and can serve as benchmarks. • The porosity and the permeability maps can be used as digital core samples for carrying out various coreflooding simulation procedures. • Analysis can be done on the entire dataset to explore how the underlying petrophysical properties affect the resulting CO_2_ saturation maps and the residual trapping relationships.

## 1 Data

**Fig. 1:**
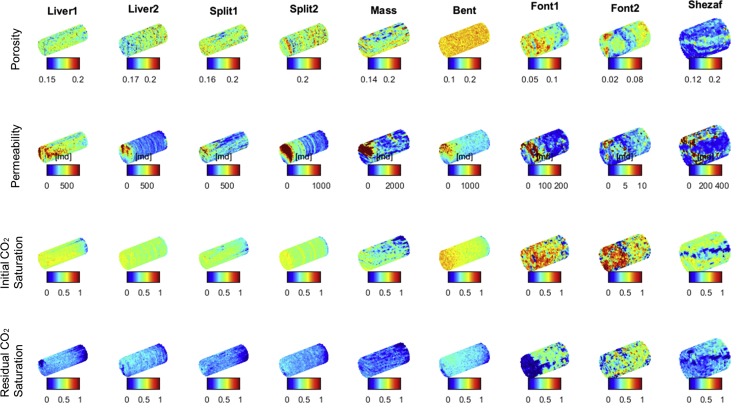
Illustration of the 3D porosity, permeability, and CO_2_ saturation maps available for the nine sandstone core samples. All voxels are coarsened and are about 2 mm × 2 mm × 2mm in size.

There are two parts to the data. The first part of the data includes Table 1, which describes the detailed experimental conditions. The second part of the data includes raw CT images and processed Matlab matrices that show 3D maps of experimental results. For each of the nine sandstone core samples, two 3D CO_2_ saturation maps, one 3D porosity map, and one 3D permeability map are shared with this article. The two CO_2_ saturation maps contain a post-drainage initial CO_2_ saturation map and a post-imbibition residual CO_2_ saturation map. Using these two CO_2_ saturation maps, residual trapping relationships can be calculated for all core samples provided. All of the 3D maps are illustrated in Fig. 1. Note that the permeability map of Fontainebleau2 is not as accurate as those of the other core samples, it is however still provided here for data completeness. For more information, see Ni et al. [1].

**Table 1:** Detailed experimental conditions for the coreflooding experiments performed on the nine sandstone rock cores.

Experiment name	Experimental temperature [°C]	Experimental pressure [psia]	Gas used	Water used	Initial scan flow rate [mL/min]	Residual scan flow rate [mL/min]	Conventional capillary number
Liver 1	50	1300	CO_2_	Pre-equilibrated deionized water	40	10	1.29E-06
Liver 2	50	1300	CO_2_	Pre-equilibrated deionized water	20	5	6.44E-07
Split 1	50	1300	CO_2_	Pre-equilibrated deionized water	20	6	7.73E-07
Split 2	50	1300	CO_2_	Pre-equilibrated deionized water	28	7	9.02E-07
Massillon	50	1300	CO_2_	Pre-equilibrated deionized water	50	15	1.93E-06
Bentheimer	50	1300	CO_2_	Pre-equilibrated deionized water	25	10	1.33E-06
Fontainebleau 1	50	1300	N_2_	Nonequilibrated deionized water	5	5	6.65E-07
Fontainebleau2	50	1300	CO_2_	Pre-equilibrated deionized water	2	0.1	1.33E-08
Shezaf	50	1300	CO_2_	Pre-equilibrated deionized water	12	1	1.38E-07

## 2 Experimental design, materials and methods

Steady-state CO_2_/water coreflooding experiments at reservoir conditions have been conducted on nine sandstone rock samples. The samples come from the Berea, Massillon, Bentheimer, Fontainebleau, and the Shezaf sandstone formations. The nine core samples also have a wide range of heterogeneity and internal features. The experiments contain both drainage and imbibition stages. The CT scans with the highest post-drainage CO_2_ saturation are selected as the initial scans and the corresponding CT scans after 100% water imbibition are selected as the residual scans to be presented with this article. Both the CO_2_ saturation maps and the porosity maps are directly obtainable through manipulating CT images, whereas the permeability maps are calculated through an extensive iterative procedure involving reservoir simulation [2], [3], [4], [5], [6]. For details regarding the experimental procedure and data processing, refer to Ni et al. [1].

Table 1 lists all the experimental conditions used for the coreflooding experiments performed on the nine sandstone cores, including experimental temperature, pressure, fluid types used, flow rates, and the conventional capillary numbers. The conventional capillary numbers reported here are achieved during 100% water imbibition stages. The following properties are used to calculate the conventional capillary number for all experiments. At a pressure of 1300 psia, CO_2_/water interfacial tension σ = 35 mN/m and water viscosity μ = 5.4843 × 10^−4^ Pa s at 50 °C [7], [8], [9], [10]. The equation for conventional capillary number is νμ/σ, where ν is the Darcy velocity.

Fig. 1 illustrates all the 3D maps provided with this data article. Each column of subplots shows the four 3D maps available for each of the nine sandstone core samples. The first row of subplots shows the porosity maps. The second row shows the permeability maps. The third row shows the initial CO_2_ saturation maps and the fourth row shows the residual CO_2_ saturation maps. All CO_2_ saturation data provided is steady-state result and the processed data has been averaged over three independent CT scans. For exact voxel sizes, refer to Ni et al. [1]. For more details on CT scan data processing, CT scan precision, and data uncertainty analysis, see Ni et al. [1] and its supplementary material.
